# Beyond aspiration: An expenditure-based evaluation of progress towards the Abuja Declaration target and implications for domestic health financing in Africa

**DOI:** 10.1371/journal.pgph.0007423

**Published:** 2026-10-09

**Authors:** Alex Olateju Adjagba, Jacob Novignon, Justice Nonvignon, Richmond Owusu, Ephrem Lemango, Helga Fogstad, Ngashi Ngongo, Jean Kaseya, George Laryea-Adjei

**Affiliations:** 1 Global Health Practice, Centre of Excellence for Child Survival and Development, UNICEF, Nairobi, Kenya; 2 Centre for Social Policy Studies, University of Ghana, Accra, Ghana; 3 School of Public Health, University of Ghana, Accra, Ghana; 4 Management Sciences for Health, Arlington, Virginia, United States of America; 5 Africa Centres for Disease Control and Prevention (Africa CDC), Addis Ababa, Ethiopia; 6 Program Division-UNICEF Headquarters, New York, United States of America; Augusta University, UNITED STATES OF AMERICA

## Abstract

In 2001, African Union (AU) member states committed to allocating 15% of their annual budget to health under the Abuja Declaration (AD). However, after more than two decades, health allocations across many countries still fall short of the target. This study uses an expenditure-based proxy for the AD to examine the likelihood and timing of attainment of the benchmark under historical fiscal trajectories.We compiled a 2002–2023 panel for 54 African countries using WHO and World Bank data. We distinguished observed, first and sustained attainment. Kaplan-Meier and Weibull models characterised time to first attainment, country-specific linear trends projected benchmark-crossing years through 2063, system GMM assessed expenditure-share persistence, and country and year fixed-effects models examined associations with primary health care (PHC) and immunisation expenditure per capita.In 2023, South Africa was the only one of 54 countries at or above the expenditure-based 15% benchmark. Five countries had attained it at least once during 2002–2023, and three sustained it for at least three consecutive years. Under the primary trend specification, which included the 51 countries with complete series, 42 (82.4%) were off track to attain the benchmark by 2063. Among the 48 countries without previous attainment, the median adjusted conditional probability of first attainment during 2023–2063 was 7.9% (IQR 4.6%–13.3%). The primary system-GMM lag coefficient was 0.821 (95% CI 0.635 to 1.006), indicating strong expenditure-share persistence. Higher government health prioritisation was associated with higher domestic government PHC expenditure per capita, while the association with total immunisation expenditure per capita was not statistically significant.Attainment of the expenditure-based Abuja benchmark remains uncommon and is unlikely to become widespread by 2063 if historical trends continue. While the 15% target remains politically useful as an accountability signal, efforts should target improving public health expenditure per capita, fiscal capacity, budget execution, financial protection and service coverage.

## Introduction

Member States of the African Union (AU) continue to face resource constraints, with health needs consistently outstripping available resources across many countries [1,2]. The burden of health financing mostly falls on individuals, with donors playing a disproportionate role, and it disproportionately affects the poor and vulnerable [3]. Strengthening domestic resource mobilization and budgetary prioritization for health is, therefore, critical for achieving universal health coverage (UHC) while addressing equity and financial protection.

In 2001, Heads of State of AU Member States committed to allocating at least 15% of annual national budgets to the health sector [4]. This commitment was necessitated by the growing burden of HIV/AIDS, tuberculosis, and Malaria at the time. While the commitment has been widely criticized for its reliance on a relative spending benchmark without accounting for differential fiscal capacity across countries, it remains a prominent, politically salient advocacy tool for health spending prioritisation on the continent [5,6]. The commitment has also drawn questions about its framing around budget allocation rather than actual spending, as incomplete budget releases and low execution rates may limit actual impact on health outcomes. Consequently, there have been legitimate concerns about whether countries will ever meet this target and how long it would take.

Moreover, relative to the time of the declaration’s adoption, health system demands across the continent have expanded significantly. In the late 1990s and early 2000s, the continent’s health landscape was dominated by HIV/AIDS, TB, and malaria, shaping mortality and morbidity substantially. In recent years, however, non-communicable diseases (NCDs) such as cardiovascular diseases, diabetes, and cancers have emerged as major threats to health systems on the continent. These have created a double disease burden across several AU Member States where infectious diseases coexist with fast-growing NCD prevalence [7]. Additionally, emerging and re-emerging threats with epidemic and pandemic potential have become routine, further burdening already strained health systems. Consequently, reliance on external financing as a major source of funding has become neither sufficient nor sustainable, reinforcing the importance of domestic budgetary commitment to health.

Studies on the AD have largely focused on descriptive assessments of historical compliance [5,6,8] or normative discussions of the appropriateness of the 15% benchmark [5]. However, there is little emphasis on forward-looking assessments of whether countries will ever meet this target under current fiscal trajectories. Such evidence is essential for assessing the feasibility of the commitment and for igniting conversations about reforming health financing across the continent. These efforts have become essential to advancing countries’ progress toward meeting the Sustainable Development Goals (SDGs) and the AU Agenda 2063, both of which clearly advocate sustainable financing and the achievement of UHC.

This study addresses this gap by assessing future progress towards the AD benchmark across AU Member States using an expenditure-based proxy. Specifically, we estimated the likelihood that countries will first attain the benchmark by 2063 and when countries may cross the threshold if historical expenditure patterns continue.

This analysis is particularly timely. Since 2019, continental leadership on domestic health financing has intensified considerably. The African Leadership Meeting (ALM) Declaration, ratified by fifty-two (52) Heads of State, established a structured commitment to increase domestic investment in health and institutionalised biennial monitoring through the Africa Scorecard on Domestic Financing for Health [9]. The second part of the ALM Declaration, the Kigali High-Level Health Financing Conference (February 2024) [10,11], convened under the auspices of President Kagame as AU Champion for Domestic Health Financing, advanced this agenda by launching Regional Health Financing Hubs and the AUDA-NEPAD Programme for Investments and Financing in Africa’s Health Sector (PIFAH) [11].

Most recently, the Africa Health Sovereignty Summit (Accra, August 2025) [12,13] produced the Accra Compact and launched the SUSTAIN initiative, a country-led framework to map health financing flows, mobilise domestic resources, and align private and philanthropic capital with national health plans [12,13]. Concurrently, Africa CDC unveiled the Africa Health Security and Sovereignty (AHSS) Agenda 33, anchored in five pillars, including innovative domestic health financing, to consolidate the continent’s shift from dependency to ownership of health systems and countermeasures.

Together, these developments signal an unprecedented level of political commitment. Yet the fundamental question our analysis addresses remains unresolved: whether the fiscal trajectories underlying health budget allocations and spending are compatible with the ambitions these initiatives embody.

## Methods

### Ethics statement and role of the funding source

The study used aggregated and anonymised secondary data publicly available, country-level secondary data and did not involve individual participants or identifiable personal information. Therefore, it did not require ethics approval

The study received no specific funding.

### Study design, data sources and variables

We constructed a country-year panel covering 2002–2023. Health-expenditure indicators were obtained from the World Health Organization Global Health Expenditure Database (WHO-GHED) [14]. Gross domestic product, under-five mortality and other development indicators came from the World Bank World Development Indicators (WDI), while government effectiveness came from the Worldwide Governance Indicators (WGI) [15,16].

The data were downloaded separately from the different sources and manually aligned in a single workbook using standardised country names and calendar year. The workbook was then imported and validated in Stata. We checked that each country-year was unique, converted source codes such as ‘..’ to missing values and applied range and consistency checks. Missing observations were retained as missing without imputation. The WHO-GHED outcome series reports identical values in consecutive years for a few countries, reflecting the source’s reporting and estimation cycle rather than annual national health accounts submissions. Across the 2002–2023 panel, 130 of 1,154 non-missing outcome observations (11.3%) were identical to the preceding year, affecting 38 of 54 countries, and 29 countries had the same reported value in 2022 and 2023 (S1 Table). These values were retained as published rather than recoded as missing, since they represent the series as reported by the source. Their implications for estimated trends and persistence are addressed in the sensitivity analyses and in the Limitations.

The primary outcome variable was domestic general government health expenditure as a percentage of general government expenditure. We classified a country as attaining the expenditure-based benchmark in a year when the observed value was at least 15%. This indicator measures executed expenditure and is not identical to the original budget-allocation commitment. Table 1 presents further details on all variables used, including the source, and definition. The variable level observation coverage is presented in S2 Table.

**Table 1:** Variable description and source.

Variable	Definition	Source
Government health-expenditure share	Domestic general government health expenditure relative to general government expenditure (% of GGE).	WHO-GHED
GDP per capita	Gross domestic product per person (US$)	World Bank WDI
External health expenditure per capita	External health expenditure per person (US$)	WHO-GHED
Under-five mortality	Probability of dying before age five (Per 1,000 live births)	World Bank WDI
Government effectiveness	Perception-based estimate of public-service quality and policy implementation	World Bank WGI
Government PHC expenditure per capita	Domestic government expenditure on primary health care per person (US$)	WHO-GHED
Total immunisation expenditure per capita	All-source immunisation expenditure share multiplied by CHE per capita (US$)	WHO-GHED
Immunisation expenditure share	Total immunisation expenditure relative to CHE (% of CHE)	WHO-GHED

Note: CHE = current health expenditure; GGE = general government expenditure; GHED = Global Health Expenditure Database; PHC = primary health care; WDI = World Development Indicators; WGI = Worldwide Governance Indicators; WHO = World Health Organization.

### Analytical samples

All 54 countries were included in the observed 2023 comparison and the historical Kaplan-Meier analysis. Fifty-one countries had complete annual outcome observations from 2002 through 2023 and formed the primary trend and persistence analysis sample. Somalia, South Sudan and Zimbabwe had shorter outcome histories and were excluded from the primary trend analysis. The adjusted Weibull model included 53 countries. South Sudan had no complete record for all four adjusted-model covariates and was therefore excluded. Conditional probability profiles were estimated for 48 countries that were included in the adjusted model and had not previously attained the benchmark. The PHC and immunisation financing models used complete available observations for each outcome. The reconciliation of each country’s inclusion across analyses is presented in S3 Table.

### Statistical analysis

The statistical analysis distinguishes observed attainment from projected progress and assesses both the persistence and financing implications of government health prioritisation. We first described current, first and sustained attainment, then used survival and country-specific trend techniques to assess historical time to first attainment and projected progress to 2063; and finally examined expenditure-share persistence and adjusted associations with PHC and immunisation expenditure. Detailed model specifications are presented in the following sub-sections.

#### Observed, first and sustained attainment.

We reported each country’s observed 2023 expenditure share, whether it attained the benchmark at least once during 2002–2023, the first attainment year, and whether it sustained attainment for at least three consecutive observed years. These measures distinguish current status from a single or repeated historical attainment.

#### Historical time to first attainment and conditional probability to 2063.

We organised annual observations as start-stop intervals from 2002 and stopped each country’s record at its first observed attainment. Countries without attainment were right censored at their last observed interval in 2023. A Kaplan-Meier curve was used to describe the historical probability that a country had not yet recorded first attainment.

To derive the conditional probability of attainment by 2063, we first fitted an adjusted Weibull proportional-hazards model using log GDP per capita, log external health expenditure per capita, under-five mortality, and government effectiveness as covariates, with country-clustered robust standard errors [17,18]. The Weibull hazard was specified as expressed in equation (1).


$$\begin{array}{c} h_{i}\left(t|X_{it}\right)=pt^{\left(p-\text{1}\right)}\text{exp}\left(X_{it}\beta \right) \end{array}$$ (1)

where $h_{i}\left(t|X_{it}\right)$ denotes the instantaneous hazard of first attainment at analysis time *t* for country *i*, conditional on not having a*t*tained previously; *p* is the Weibull shape parameter, *X* is the vector of country-year covariates, and β is the corresponding vector of coefficients.

For countries that remained at risk at the end of 2023, we calculated the probability of first attainment between 2023 and 2063 conditional on non-attainment through 2023:


$$\begin{array}{c} P\left(T_{i}\leq \text{2}\text{0}\text{6}\text{3}|T_{i}>\text{2}\text{0}\text{2}\text{3},X_{i}\right)=\text{1}-\frac{S_{i}\left(\text{2}\text{0}\text{6}\text{3}|X_{i}\right)}{S_{i}\left(\text{2}\text{0}\text{2}\text{3}|X_{i}\right)} \end{array}$$ (2)

where *T*_*i*_ denotes the calendar year of first attainment for country *i*, $S_{i}\left(t|X_{i}\right)$ denotes the fitted Weibull probability of remaining without first attainment through year *t*, and *X*_*i*_ represents the included last observed coun*t*ry-level covariates.

Predictions held each country’s latest complete covariate profile constant through 2063. Forty-six profiles used 2023 values; Eritrea used 2011, and Seychelles used 2020, the latest years with complete prediction covariates. We used 2,000 country-cluster bootstrap replications, resampling countries with replacement and refitting the adjusted Weibull model in each replication (seed 24042026). The 95% percentile bootstrap intervals were defined by the 2.5th and 97.5th percentiles of the successful country-specific predictions.

#### Country-specific trend projections.

For each country, we estimated an ordinary least-squares model of the expenditure share as a linear function of year as expressed in equation (3).


$$\begin{array}{c} H_{it}=\alpha_{i}+\beta_{i}{year}_{t}+\varepsilon_{it} \end{array}$$ (3)

where $H_{it}$ denotes the government health-expenditure share for country *i* in year *t*, $\alpha_{i}$ is the country-specific intercep*t*, $\beta_{i}$ is the estimated annual change in the expenditure share, *year*_*i*_ denotes the calendar year, and $\varepsilon_{it}$ is the error term.

For countries below 15% in 2023 with a positive fitted slope, the point-estimate crossing year was calculated as the first year in which the fitted share reached 15%. Countries were classified as already attained when the observed 2023 value was at least 15%; on track when the projected crossing year was between 2024 and 2048; slow progress when it was 2049–2063; and off track when the fitted trend did not reach 15% by 2063.

We drew 2,000 coefficient vectors from each country’s estimated coefficient distribution. The percentage of draws crossing by 2063 provided a model-based crossing probability, while the 2.5th and 97.5th percentiles among draws that crossed provided a conditional interval for the crossing year. These intervals capture coefficient uncertainty under the fitted linear model.

The primary analysis used all annual observations from 2002-2023 for the 51 countries with complete outcome histories. Alternative period sensitivity analyses used (i) all available 2002–2023 non-missing observations for 54 countries (including Somalia, South Sudan and Zimbabwe with shorter histories), (ii) a recent 2010–2023 period and (iii) a pre-COVID-19 period ending in 2019 [19]. The analytical sample varied across specifications because of differences in outcome coverage. The primary and pre-COVID-19 specifications used a complete observation window and therefore included the 51 countries with uninterrupted outcome histories. The available data and recent-period specifications estimate trends from all non-missing observations within the relevant estimation windows and therefore retained all 54 countries. The denominator for each classification is the number of countries estimated in that specification. Because exact repeated annual values reduce the within-country variation available for estimating country-specific trends, we conducted additional sensitivity analyses within the primary 51-country sample, requiring at least 10 or at least 15 distinct annual outcome values across the 22 years. These restrictions retained 50 and 46 countries, respectively, while retaining repeated country-years among eligible countries. Results are reported in S4 Table (Country-specific trend classification after excluding countries with limited outcome variation).

#### Expenditure-share persistence.

We assessed persistence in government health prioritisation using a dynamic-panel model specification expressed in equation (4).


$$\begin{array}{c} H_{it}=\rho H_{i,t-\text{1}}+\beta^{\prime }X_{it}+\lambda_{t}+\mu_{i}+\varepsilon_{it} \end{array}$$ (4)

where ρ is the lagged expenditure-share coefficient; X_it_ contains log GDP per capita, log external health expenditure per capita, under-five mortality, and government effectiveness; λ_t_ denotes year effects; and μ_i_ denotes unobserved time-invariant country characteristics. The coefficient ρ measures year-to-year persistence in the expenditure share.

The primary model used a two-step system generalised method of moments (GMM) [20,21]. The lagged expenditure share was treated as predetermined and instrumented. The covariates and year indicators entered as standard instruments. We used finite-sample-corrected robust standard errors [22] and restricted the lag range and collapsed the instrument matrix to reduce instrument proliferation [23,24]. We reported Arellano-Bond tests for first- and second-order serial correlation, the Hansen test of overidentifying restrictions, and the number of instruments relative to country groups. Sensitivity analyses included one-step difference GMM, two-step system GMM restricted to lag 2, a pooled dynamic model with year effects and a country-and-year fixed-effects model.

#### Associations with PHC and immunisation expenditure.

We also estimated separate log-linear fixed-effects models to determine the association between PHC and immunisation expenditure and government health expenditure. Equation (5) provides the panel model specification.


$$\begin{array}{c} n\left(Y_{it}\right)=\beta H_{it}+\alpha_{i}+\lambda_{t}+\varepsilon_{it} \end{array}$$ (5)

where Y_it_ was domestic government PHC expenditure per capita or total immunisation expenditure per capita, H_it_ was the government health-expenditure share, αᵢ denoted country fixed effects, and λ_t_ denoted year fixed effects. Country fixed effects accounted for time-invariant country characteristics, year effects captured common annual shocks, and standard errors were clustered by country to account for repeated observations and within-country correlation. Coefficients were converted to percentage changes and represent the adjusted within-country percentage difference in expenditure per capita associated with a one-percentage-point increase in the government health-expenditure share.

#### Software, statistical uncertainty and reproducibility.

Analyses were conducted in Stata 17 [25]. Statistical tests were two-sided and 95% confidence intervals are reported. Bootstrap and coefficient-simulation procedures used 2,000 replications and random seed 24042026.

## Results

### Financing composition and observed 2023 attainment

Across the study period, private sources remained the largest component of current health expenditure on average, although the balance between domestic government, external and private financing varied over time (Fig 1). In 2023, South Africa was the only one of 54 countries at or above the expenditure-based 15% benchmark, with a value of 16.89% (Fig 2). Namibia recorded 14.99% and was therefore classified below the threshold.

**Fig 1:**
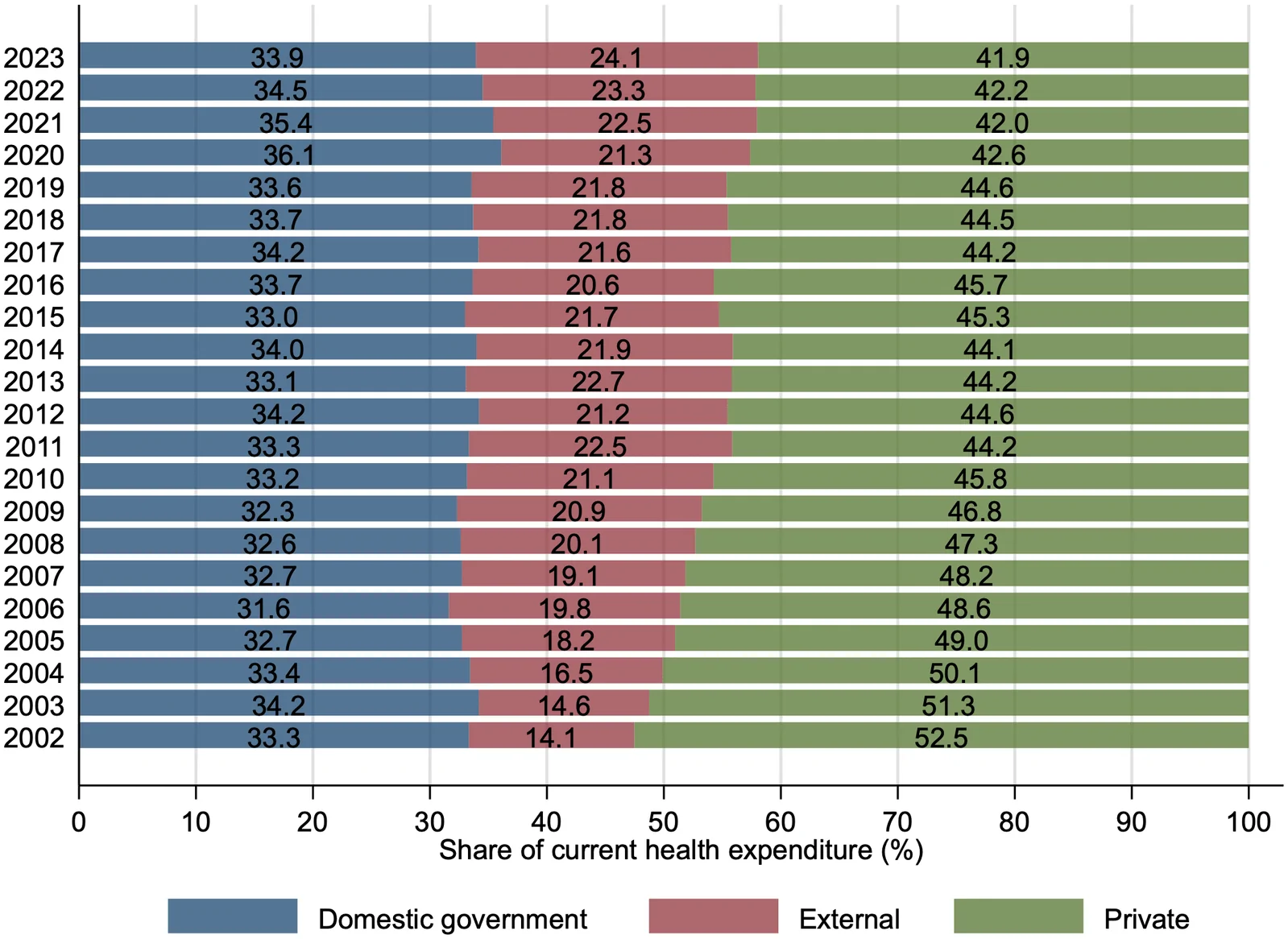
Average health-financing composition across African countries, 2002-2023.

**Fig 2:**
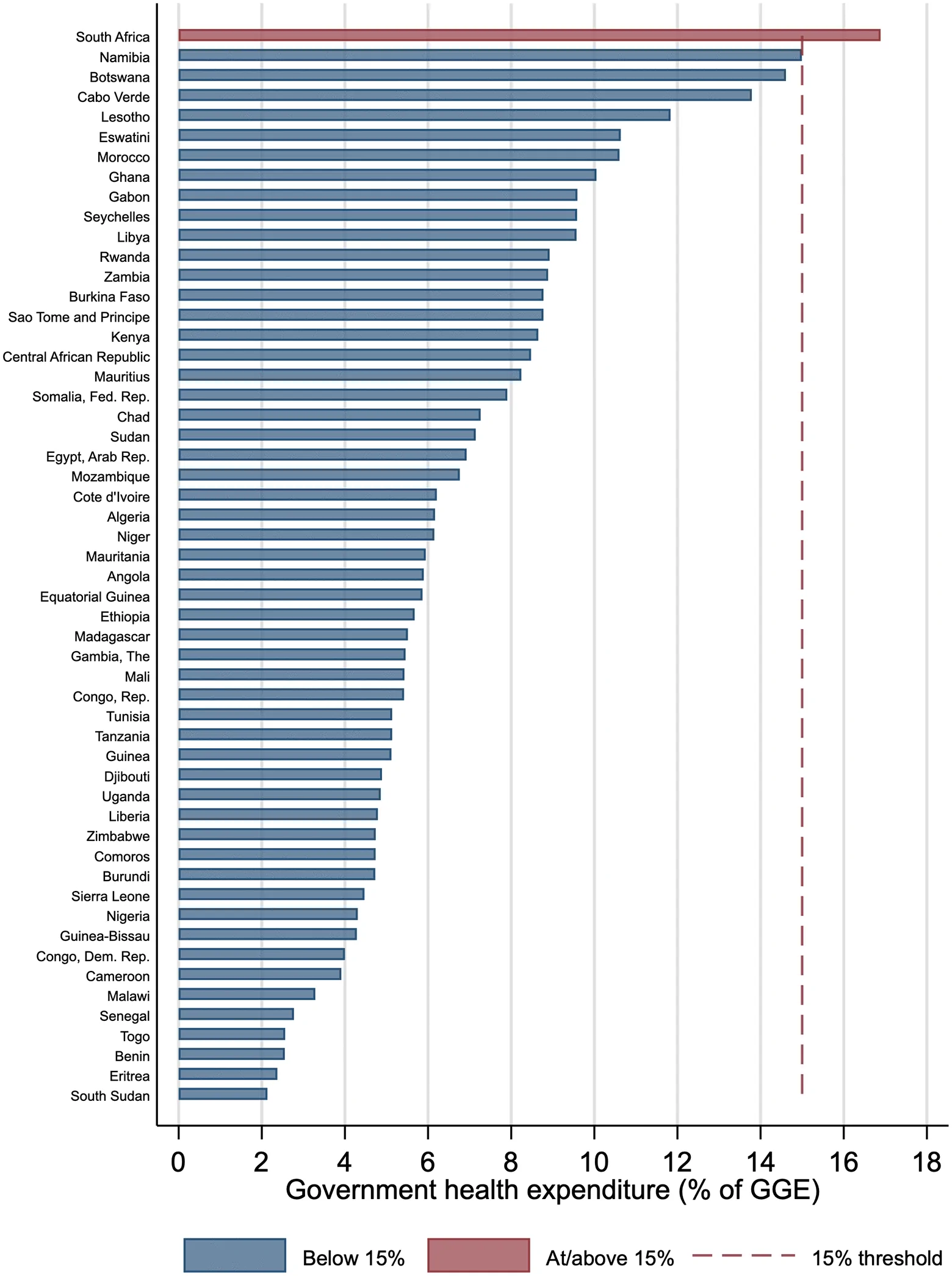
Government health-expenditure shares across 54 African countries, 2023.

### Historical first and sustained attainment

Five countries recorded at least one year at or above 15% during 2002–2023. These were Namibia in 2002, Chad in 2004, Madagascar in 2010, South Africa in 2015 and Cabo Verde in 2022. Namibia, Madagascar and South Africa sustained attainment for at least three consecutive years (S3 Table). By 2023, only South Africa remained at or above the benchmark. The Kaplan-Meier survivor probability in 2023 was 0.904, confirming that first recorded attainment was uncommon (Fig 3).

**Fig 3:**
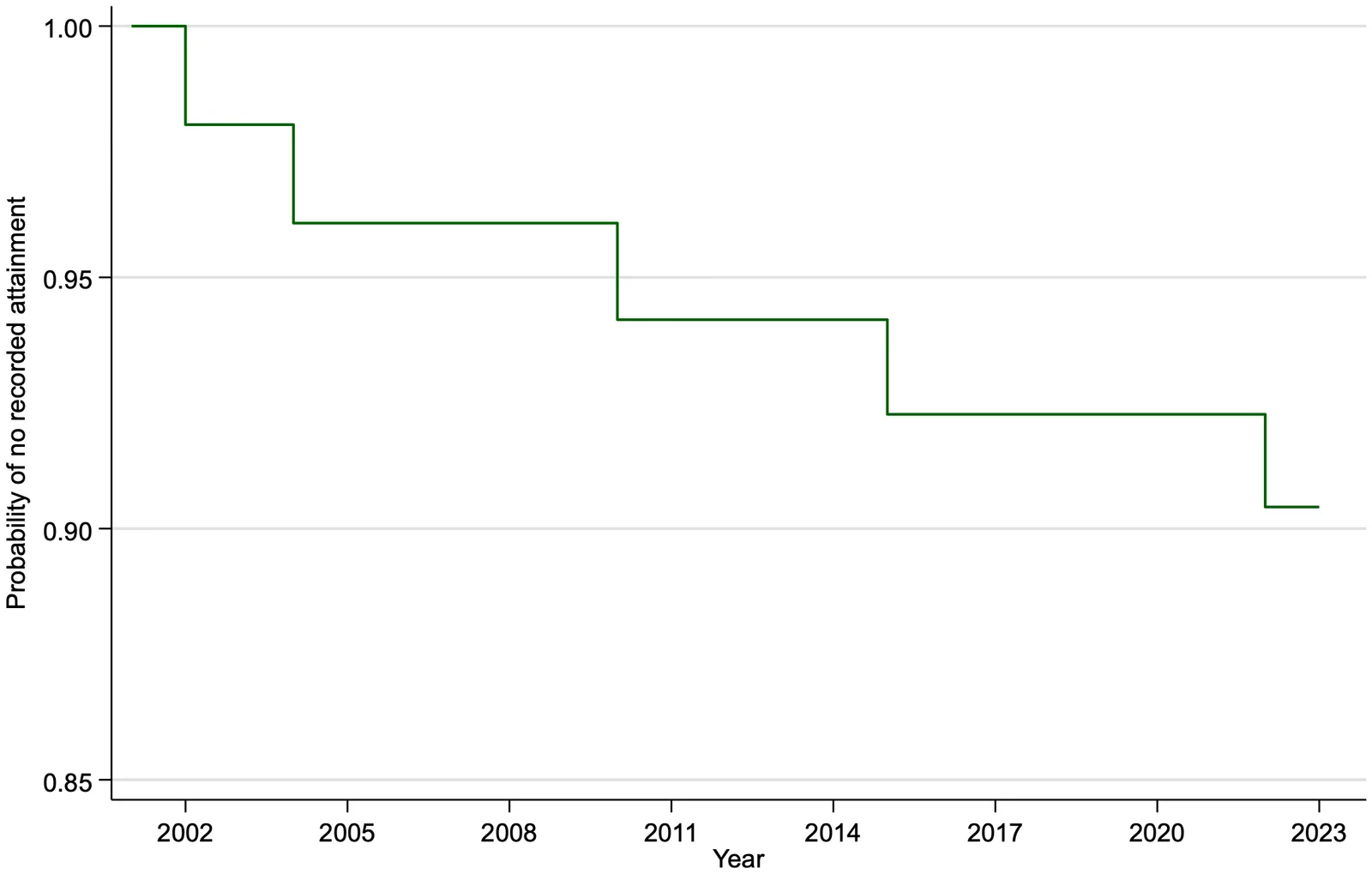
Time to first recorded attainment of the Abuja expenditure benchmark, 2002-2023.

### Conditional probabilities of first attainment

Fig 4 reports adjusted conditional probabilities of first attainment during 2023–2063 for 48 countries that had not previously attained the benchmark and had complete adjusted-model covariate data. The median probability was 7.9% (IQR 4.6%- 13.3%). One of 48 countries had a point estimate below 1%, while 42 of 48 had estimates below 20%. However, the country-cluster bootstrap intervals were wide, reflecting the fact that only five first-attainment events informed the adjusted Weibull model (S5 Table). The full results of the adjusted model are preented in S6 Table.

**Fig 4:**
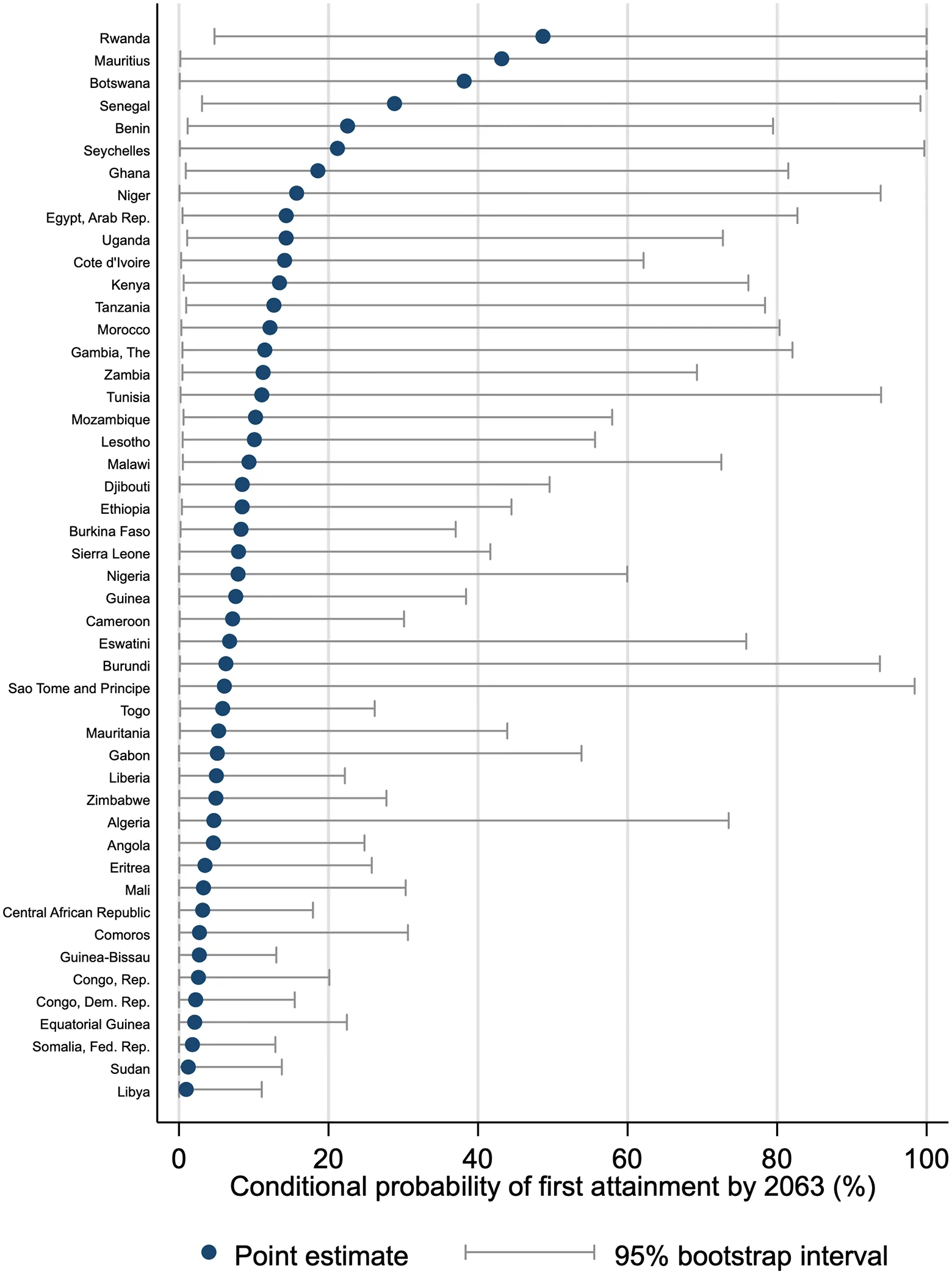
Conditional probabilities of first Abuja-benchmark attainment by 2063.

### Country-specific trend projections

Under the primary 2002–2023 complete-panel specification, 1 of 51 countries had already attained the benchmark in 2023, 4 (7.8%) were on track to cross by 2048, 4 (7.8%) showed slow progress with projected crossing during 2049–2063, and 42 (82.4%) were off track by 2063 (Fig 5). Point-estimate crossing years ranged from 2026 for Botswana to 2058 for Mauritius. The point estimates crossing years and their conditional 95% simulation intervals are reported in S7 Table.

**Fig 5:**
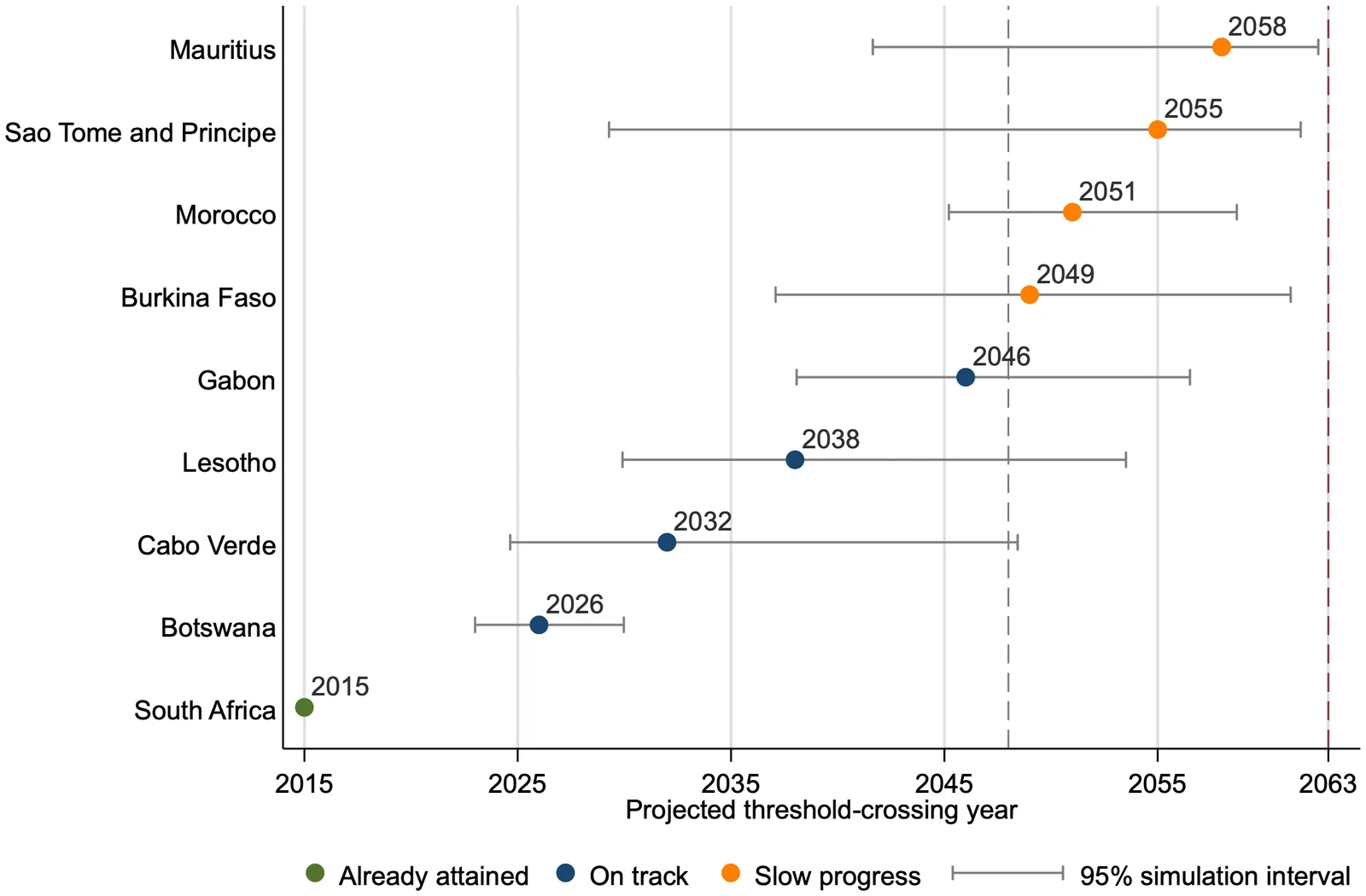
Projected Abuja-benchmark attainment years under country-specific historical trends.

The conclusion that most countries were off track was maintained across the alternative specifications, each estimated on its own analytical sample. The available data 2002–2023 analysis classified 45 of 54 countries (83.3%) as off track; the recent 2010–2023 analysis classified 37 of 54 (68.5%); and the pre-COVID-19 common-period analysis classified 40 of 51 (78.4%) (S8 Table). The conclusion was also consistent in the additional sensitivity analysis to improve outcome variation by focusing on countries with distinct annual outcome values (S4 Table).

### Expenditure-share persistence

The two-step system-GMM lag coefficient was 0.82 (95% CI: 0.635-1.006; p < 0.001), indicating strong year-to-year persistence in the government health expenditure share (Fig 6). The difference-GMM and restricted-instrument system-GMM estimates were 0.88 and 0.84, respectively, with wider confidence intervals, so all three estimators produced lag coefficients above 0.8. The confidence intervals are wide and the upper bound of the primary estimate is consistent with near-unit-root behaviour, so the coefficient should be read as evidence of slow-moving expenditure prioritisation rather than as a precise convergence parameter. A sensitivity estimate excluding country-years with values identical to the preceding year is reported in S9 Table. This estimate remained statistically different from zero, indicating substantial persistence. However, its confidence interval included one, suggesting that complete persistence could not be rejected. The full model coefficients results are reported in S10 Table and the dynamic-panel diagnostics are reported in S11 Table.

**Fig 6:**
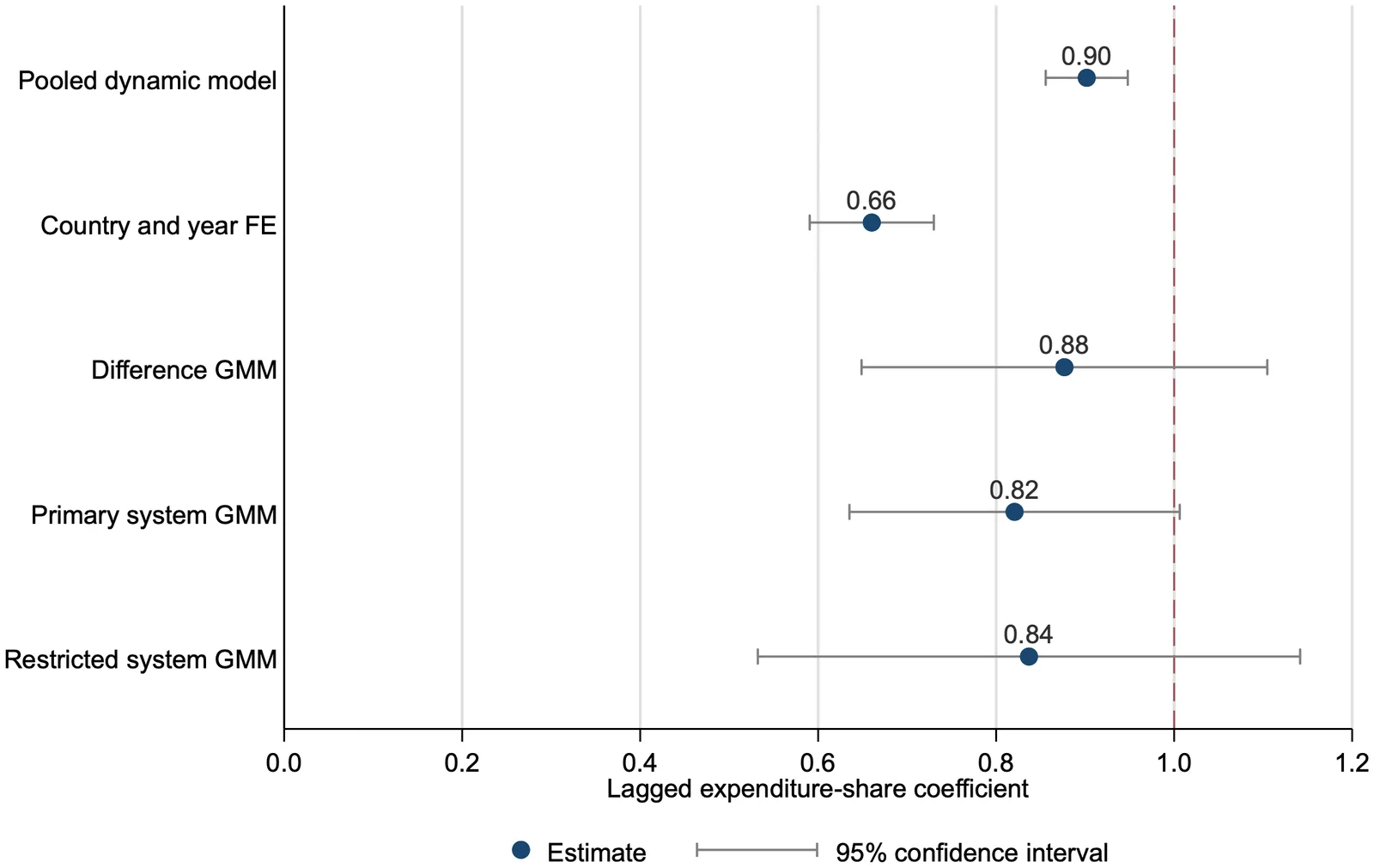
Adjusted associations of government health prioritisation with PHC and immunisation expenditure.

### Associations with PHC and immunisation expenditure

In the country-and-year fixed-effects model, a one-percentage-point increase in the government health-expenditure share was associated with 15.3% higher domestic government PHC expenditure per capita (95% CI: 10.3%-20.4%; p < 0.001) (Fig 7). The corresponding estimate for total immunization expenditure per capita was 5.2% but was not statistically significant (95% CI: -7.9%-20.3%; p = 0.446). The detailed model estimates and additional parameters are reported in S12 Table. Using immunisation expenditure as a share of CHE instead of immunization expenditure per capita did not change this conclusion (S13 Table)

**Fig 7:**
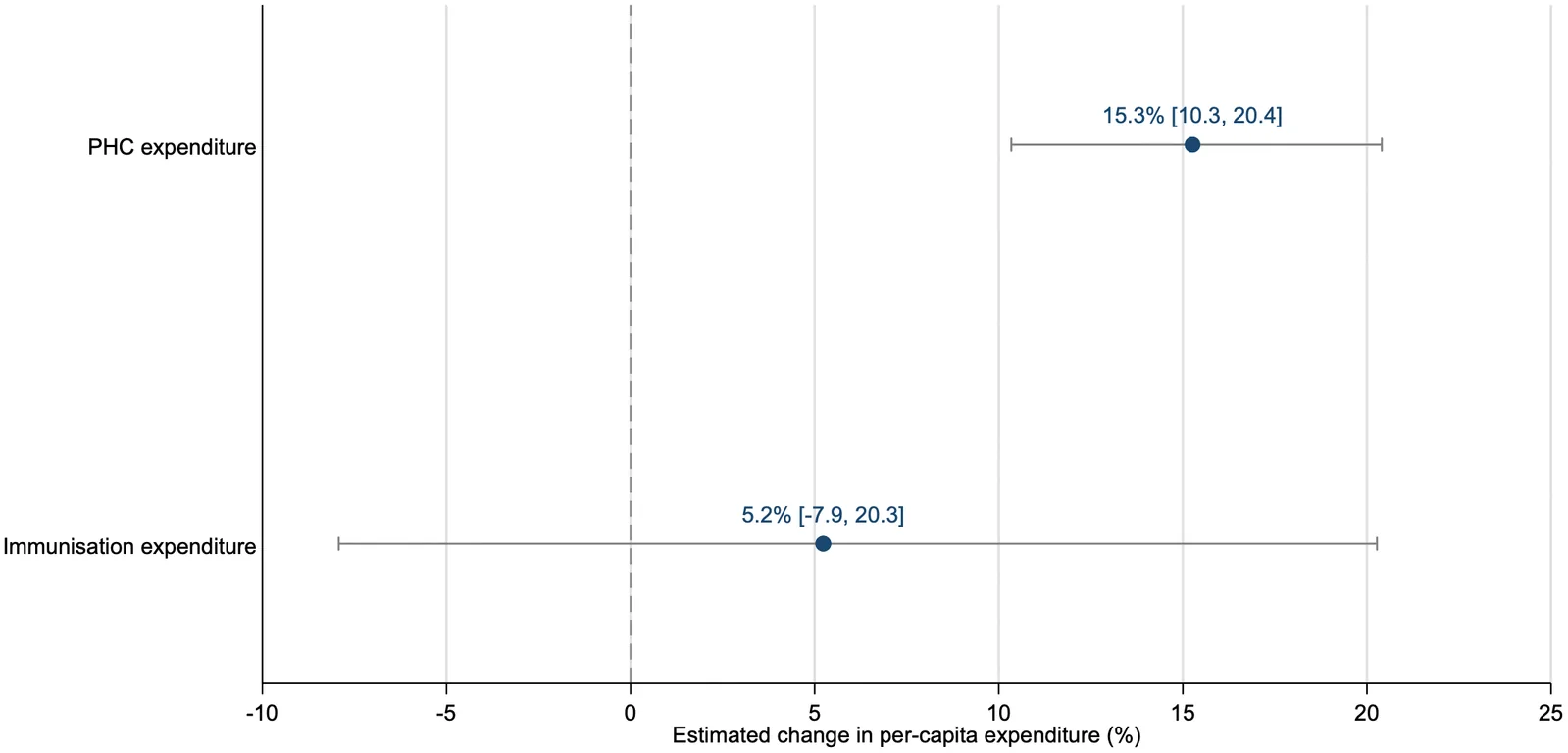
Persistence of government health-expenditure shares across model specifications.

## Discussion

This study provides a forward-looking assessment of an expenditure-based proxy for the Abuja commitment. The principal finding is that attainment remains uncommon and is unlikely to become widespread by 2063 if historical relationships persist. Only South Africa was at or above 15% in 2023, five countries had ever attained it during the study period, and only three sustained attainments for at least three consecutive years. In the primary trend model, 82.4% of countries were off track through 2063.

These results are consistent with earlier descriptive assessments showing limited and episodic attainment of the Abuja benchmark [8,26]. The distinction between first, sustained and current attainment is important. A single historical crossing may reflect a temporary fiscal or expenditure change and does not establish that prioritisation was maintained. The historical Kaplan-Meier analysis and the sparse number of first-attainment events reinforce this point.

The trend and persistence analyses provide complementary evidence. Country-specific projections indicate that the continuation of average 2002–2023 trends would leave most countries below the benchmark in 2063. The system-GMM coefficient shows that expenditure shares are strongly path-dependent. It indicates that countries’ expenditure prioritisation tends to change slowly, strengthening the case for policy changes capable of creating structural breaks from historical trajectories.

The findings do not imply that the Abuja benchmark should simply be abandoned. Its simplicity and political visibility continue to make it useful for advocacy and accountability. However, a relative expenditure share is a measure of prioritisation rather than financing adequacy. A country can devote 15% of a small government budget to health and still spend too little per person to provide essential services, while another may remain below 15% yet achieve higher absolute public spending and stronger coverage. The findings therefore call for complementary efforts that improve public health expenditure per capita, government revenue and fiscal capacity, budget execution, financial protection, service coverage and equity. Our analysis does not identify a single replacement threshold, rather, it supports a broader monitoring framework that reflects country context and financing outcomes [27,28].

The service-financing results add an allocative dimension. Higher government health prioritisation was associated with higher domestic government PHC expenditure per capita, consistent with the importance of pooled public financing for system-oriented services [29]. By contrast, the estimate for total immunisation expenditure per capita was positive but statistically insignificant. Programme-based external financing may partly weaken the relationship between government prioritisation and total immunisation spending, but the available indicator combines all financing sources and cannot fully isolate this mechanism [30]. However, broader evidence distinguishes system-wide investments from vertically financed programmes, highlighting that increases in overall health budget shares do not automatically translate into sustained domestic financing for programmes that already heavily depend on external financing. For instance, Sakala et al. provide evidence from Malawi showing that vertical programmes (such as immunisation) depend almost entirely on external funding, often outside routine government budgets. Based on these findings, we propose that integrating immunization programmes fully into health systems may facilitate increasing sustainable domestic financing for immunisation.

Recent continental initiatives provide an institutional basis for moving beyond status-quo trajectories. The Africa Scorecard, Regional Health Financing Hubs, SUSTAIN and the Africa Health Security and Sovereignty Agenda place greater emphasis on domestic ownership, joint monitoring and alignment of financing with national health plans [9,11–13,31,32]. Our results suggest that these mechanisms will be most consequential when they support country-level changes in revenue mobilisation, medium-term budgeting, budget execution, public financial management and expenditure tracking. Political commitments and monitoring can create pressure for change, but operational reforms determine whether that pressure alters actual expenditure.

Ultimately, these projections provide conditional scenarios of the prospects of domestic health financing across AU member states. Deliberate reprioritisation, revenue reform, improved execution, debt relief, economic growth or changes in external financing could shift country trajectories. Conversely, macroeconomic shocks and declining external support could slow progress. The value of the projections is to quantify the implications of continuing historical patterns and to show the scale of departure required if most countries are to attain the expenditure benchmark within the agenda 2063 horizon.

### Limitations

This study has some limitations that are worth mentioning. First, the primary outcome is an expenditure-based proxy for the Abuja benchmark and differs from the government budget-allocation measure specified in the original Declaration. It therefore measures actual expenditure prioritisation rather than formal budget commitments.

Second, the WHO-GHED outcome series reports identical values in consecutive years for a substantial share of country-years (S1 Table). This reflects the source’s reporting cycle rather than an analytical decision. Exact consecutive-year repeats reduce observed within-country variation and may influence estimated trends and persistence. We therefore assessed the robustness of the findings using restrictions based on the number of distinct annual values and, for persistence, a sensitivity analysis excluding repeated observations. The results were consistent across all the sensitivity analyses (S4 and S9 Tables). However, the point estimates for both the trend classifications and the persistence coefficient should be interpreted with this feature of the source data in mind.

Third, the survival estimates were informed by only five first-attainment events. The conditional probabilities should therefore be considered exploratory, particularly because several country-specific confidence intervals were wide.

Fourth, the trend projections extrapolate historical linear relationships over an extended period and cannot capture future policy reforms, macroeconomic shocks, health emergencies or nonlinear changes in expenditure patterns.

Finally, the PHC and immunisation analyses were observational and remain subject to time-varying confounding and reverse causation despite including country and year fixed effects.

## Conclusion

Across African countries, observed and projected expenditure patterns indicate that attainment of the expenditure-based proxy for the Abuja benchmark has been rare and, under historical trends, most countries are unlikely to attain it by 2063. These projections are, however, status-quo scenarios, and deliberate fiscal and health-financing reforms can alter country trajectories. The 15% benchmark can continue to serve as an important political accountability signal, but it should not be used alone to assess health-financing adequacy. Monitoring should combine budget and expenditure shares with per-capita public health spending, fiscal capacity, budget execution, financial protection and service coverage. The central policy implication is therefore not simply to pursue a single threshold, but to strengthen the institutions that convert sustained fiscal prioritisation into predictable, efficiently executed and equitable health financing.

## Supporting information

S1 Table Exact consecutive-year repeats and distinct annual outcome values by country. (DOCX)

S2 Table Variable-level coverage. (DOCX)

S3 Table Country-level reconciliation of outcome coverage, attainment and analytical inclusion. (DOCX)

S4 Table Country-specific trend classification after excluding countries with limited outcome variation. (DOCX)

S5 Table Weibull model sample and fit summary. (DOCX)

S6 Table Adjusted Weibull proportional-hazards estimates. (DOCX)

S7 Table Countries projected to cross the benchmark by 2063 in the primary specification. (DOCX)

S8 Table Sensitivity of trend classifications to outcome coverage and estimation period. (DOCX)

S9 Table System-GMM persistence estimates after restricting outcome variation and excluding exact consecutive-year repeats. (DOCX)

S10 Table Full substantive coefficients from the GMM specifications. (DOCX)

S11 Table Lagged expenditure-share estimates and dynamic-panel diagnostics. (DOCX)

S12 Table Country- and year-fixed-effects estimates for per-capita PHC and immunisation expenditure. (DOCX)

S13 Table Sensitivity analysis using immunisation expenditure as a percentage of CHE. (DOCX)

S1 Data Data. (XLSX)

## Data sharing statement

All data underlying the findings of this study are publicly available and are provided in full as supporting information. The harmonised country-year analytical dataset for 54 African countries, 2002–2023, is provided as S1 Data. The original sources are the WHO Global Health Expenditure Database (https://apps.who.int/nha/database), the World Bank World Development Indicators (https://databank.worldbank.org), and the World Bank Worldwide Governance Indicators [15] (https://www.worldbank.org/en/publication/worldwide-governance-indicators). All analyses were conducted in Stata using standard published commands, which are fully specified in the Methods Section. The Stata do-files are available from the corresponding author on request. No restrictions apply to the data.
