# Supplementary material for: Beyond aspiration: An expenditure-based evaluation of progress towards the Abuja Declaration target and implications for domestic health financing in Africa
Source: PLOS Glob Public Health. 2026 Oct 9;6(10):e0007423. doi: 10.1371/journal.pgph.0007423 (PMC13652233; doi:10.1371/journal.pgph.0007423)
Supplement: Supplementary file 1 [file pgph.0007423.s001.docx]

**S1 Table. Exact consecutive-year repeats and distinct annual outcome values by country.**

| **Country** | **Non-missing annual values** | **Eligible annual comparisons** | **Distinct annual values** | **Exact repeated country-years** | **Any exact repeat** | **Identical values in 2022 and 2023** |
| --- | --- | --- | --- | --- | --- | --- |
| Algeria | 22 | 21 | 22 | 0 | No | No |
| Angola | 22 | 21 | 21 | 1 | Yes | No |
| Benin | 22 | 21 | 21 | 1 | Yes | Yes |
| Botswana | 22 | 21 | 15 | 7 | Yes | Yes |
| Burkina Faso | 22 | 21 | 22 | 0 | No | No |
| Burundi | 22 | 21 | 19 | 3 | Yes | Yes |
| Cabo Verde | 22 | 21 | 22 | 0 | No | No |
| Cameroon | 22 | 21 | 20 | 2 | Yes | Yes |
| Central African Republic | 22 | 21 | 21 | 1 | Yes | Yes |
| Chad | 22 | 21 | 20 | 2 | Yes | Yes |
| Comoros | 22 | 21 | 20 | 2 | Yes | Yes |
| Congo, Dem. Rep. | 22 | 21 | 14 | 8 | Yes | No |
| Congo, Rep. | 22 | 21 | 21 | 1 | Yes | No |
| Cote d'Ivoire | 22 | 21 | 21 | 1 | Yes | Yes |
| Djibouti | 22 | 21 | 22 | 0 | No | No |
| Egypt, Arab Rep. | 22 | 21 | 21 | 1 | Yes | No |
| Equatorial Guinea | 22 | 21 | 22 | 0 | No | No |
| Eritrea | 22 | 21 | 6 | 16 | Yes | Yes |
| Eswatini | 22 | 21 | 21 | 1 | Yes | Yes |
| Ethiopia | 22 | 21 | 20 | 2 | Yes | Yes |
| Gabon | 22 | 21 | 18 | 4 | Yes | Yes |
| Gambia, The | 22 | 21 | 11 | 11 | Yes | No |
| Ghana | 22 | 21 | 22 | 0 | No | No |
| Guinea | 22 | 21 | 21 | 1 | Yes | Yes |
| Guinea-Bissau | 22 | 21 | 13 | 9 | Yes | No |
| Kenya | 22 | 21 | 21 | 1 | Yes | Yes |
| Lesotho | 22 | 21 | 22 | 0 | No | No |
| Liberia | 22 | 21 | 12 | 10 | Yes | Yes |
| Libya | 22 | 21 | 22 | 0 | No | No |
| Madagascar | 22 | 21 | 21 | 1 | Yes | Yes |
| Malawi | 22 | 21 | 21 | 1 | Yes | Yes |
| Mali | 22 | 21 | 22 | 0 | No | No |
| Mauritania | 22 | 21 | 21 | 1 | Yes | Yes |
| Mauritius | 22 | 21 | 22 | 0 | No | No |
| Morocco | 22 | 21 | 22 | 0 | No | No |
| Mozambique | 22 | 21 | 20 | 2 | Yes | No |
| Namibia | 22 | 21 | 22 | 0 | No | No |
| Niger | 22 | 21 | 22 | 0 | No | No |
| Nigeria | 22 | 21 | 20 | 2 | Yes | Yes |
| Rwanda | 22 | 21 | 20 | 2 | Yes | No |
| Sao Tome and Principe | 22 | 21 | 22 | 0 | No | No |
| Senegal | 22 | 21 | 21 | 1 | Yes | Yes |
| Seychelles | 22 | 21 | 17 | 5 | Yes | Yes |
| Sierra Leone | 22 | 21 | 16 | 6 | Yes | Yes |
| Somalia, Fed. Rep. | 11 | 10 | 11 | 0 | No | No |
| South Africa | 22 | 21 | 16 | 6 | Yes | Yes |
| South Sudan | 7 | 6 | 2 | 5 | Yes | No |
| Sudan | 22 | 21 | 22 | 0 | No | No |
| Tanzania | 22 | 21 | 19 | 3 | Yes | Yes |
| Togo | 22 | 21 | 20 | 2 | Yes | Yes |
| Tunisia | 22 | 21 | 19 | 3 | Yes | Yes |
| Uganda | 22 | 21 | 21 | 1 | Yes | Yes |
| Zambia | 22 | 21 | 21 | 1 | Yes | Yes |
| Zimbabwe | 14 | 13 | 11 | 3 | Yes | Yes |

**Notes:** Across 1154 non-missing outcome observations, 130 (11.3%) exactly repeated the immediately preceding year's value. Repeats affected 38 of 54 countries, including 29 countries with identical values in 2022 and 2023. The first observed year and observations without a non-missing value in the immediately preceding calendar year were not eligible to be classified as repeats
