# Supplementary material for: Beyond aspiration: An expenditure-based evaluation of progress towards the Abuja Declaration target and implications for domestic health financing in Africa
Source: PLOS Glob Public Health. 2026 Oct 9;6(10):e0007423. doi: 10.1371/journal.pgph.0007423 (PMC13652233; doi:10.1371/journal.pgph.0007423)
Supplement: Supplementary file 2 [file pgph.0007423.s002.docx]

**S2 Table. Variable-level coverage.**

| **Variable** | **Possible country-years** | **Non-missing observations** | **Missing observations** | **Countries with data (n)** | **First year** | **Last year** |
| --- | --- | --- | --- | --- | --- | --- |
| Domestic general government health expenditure (% of GGE) | 1188 | 1154 | 34 | 54 | 2002 | 2023 |
| GDP per capita (current US$) | 1188 | 1162 | 26 | 54 | 2002 | 2023 |
| External health expenditure per capita (current US$) | 1188 | 1153 | 35 | 54 | 2002 | 2023 |
| Under-five mortality rate (per 1,000 live births) | 1188 | 1188 | 0 | 54 | 2002 | 2023 |
| Government effectiveness index | 1188 | 1179 | 9 | 54 | 2002 | 2023 |
| Government revenue (% of GDP) | 1188 | 814 | 374 | 37 | 2002 | 2023 |
| Current health expenditure per capita (current US$) | 1188 | 1154 | 34 | 54 | 2002 | 2023 |
| Domestic government PHC expenditure per capita (US$) | 1188 | 211 | 977 | 45 | 2016 | 2023 |
| Immunisation expenditure (% of CHE) | 1188 | 199 | 989 | 46 | 2016 | 2023 |
| Total immunisation expenditure per capita (US$) | 1188 | 199 | 989 | 46 | 2016 | 2023 |
| Life expectancy at birth (years) | 1188 | 1188 | 0 | 54 | 2002 | 2023 |

*Notes: The data contains 1,188 possible country-year records (54 countries x 22 years).*
