# Supplementary material for: Beyond aspiration: An expenditure-based evaluation of progress towards the Abuja Declaration target and implications for domestic health financing in Africa
Source: PLOS Glob Public Health. 2026 Oct 9;6(10):e0007423. doi: 10.1371/journal.pgph.0007423 (PMC13652233; doi:10.1371/journal.pgph.0007423)
Supplement: Supplementary file 3 [file pgph.0007423.s003.docx]

**S3 Table. Country-level reconciliation of outcome coverage, attainment and analytical inclusion.**

| **Country** | **Outcome coverage (years)** | **Government health expenditure, 2023 (% of GGE)** | **Prior attainment** | **Sustained attainment (≥3 consecutive years)** | **Primary trend status** | **Included in adjusted Weibull model** | **Included in conditional probability analysis** | **Covariate profile year** |
| --- | --- | --- | --- | --- | --- | --- | --- | --- |
| Algeria | 2002-2023 | 6.17 | No | No | Yes - Off track | Yes | Yes | 2023 |
| Angola | 2002-2023 | 5.90 | No | No | Yes - Off track | Yes | Yes | 2023 |
| Benin | 2002-2023 | 2.55 | No | No | Yes - Off track | Yes | Yes | 2023 |
| Botswana | 2002-2023 | 14.61 | No | No | Yes - On track | Yes | Yes | 2023 |
| Burkina Faso | 2002-2023 | 8.78 | No | No | Yes - Slow progress | Yes | Yes | 2023 |
| Burundi | 2002-2023 | 4.73 | No | No | Yes - Off track | Yes | Yes | 2023 |
| Cabo Verde | 2002-2023 | 13.79 | Yes (2022) | No | Yes - On track | Yes | No |  |
| Cameroon | 2002-2023 | 3.91 | No | No | Yes - Off track | Yes | Yes | 2023 |
| Central African Republic | 2002-2023 | 8.48 | No | No | Yes - Off track | Yes | Yes | 2023 |
| Chad | 2002-2023 | 7.26 | Yes (2004) | No | Yes - Off track | Yes | No |  |
| Comoros | 2002-2023 | 4.74 | No | No | Yes - Off track | Yes | Yes | 2023 |
| Congo, Dem. Rep. | 2002-2023 | 4.00 | No | No | Yes - Off track | Yes | Yes | 2023 |
| Congo, Rep. | 2002-2023 | 5.42 | No | No | Yes - Off track | Yes | Yes | 2023 |
| Cote d'Ivoire | 2002-2023 | 6.21 | No | No | Yes - Off track | Yes | Yes | 2023 |
| Djibouti | 2002-2023 | 4.89 | No | No | Yes - Off track | Yes | Yes | 2023 |
| Egypt, Arab Rep. | 2002-2023 | 6.93 | No | No | Yes - Off track | Yes | Yes | 2023 |
| Equatorial Guinea | 2002-2023 | 5.87 | No | No | Yes - Off track | Yes | Yes | 2023 |
| Eritrea | 2002-2023 | 2.37 | No | No | Yes - Off track | Yes | Yes | 2011 |
| Eswatini | 2002-2023 | 10.64 | No | No | Yes - Off track | Yes | Yes | 2023 |
| Ethiopia | 2002-2023 | 5.68 | No | No | Yes - Off track | Yes | Yes | 2023 |
| Gabon | 2002-2023 | 9.59 | No | No | Yes - On track | Yes | Yes | 2023 |
| Gambia, The | 2002-2023 | 5.46 | No | No | Yes - Off track | Yes | Yes | 2023 |
| Ghana | 2002-2023 | 10.05 | No | No | Yes - Off track | Yes | Yes | 2023 |
| Guinea | 2002-2023 | 5.12 | No | No | Yes - Off track | Yes | Yes | 2023 |
| Guinea-Bissau | 2002-2023 | 4.29 | No | No | Yes - Off track | Yes | Yes | 2023 |
| Kenya | 2002-2023 | 8.65 | No | No | Yes - Off track | Yes | Yes | 2023 |
| Lesotho | 2002-2023 | 11.83 | No | No | Yes - On track | Yes | Yes | 2023 |
| Liberia | 2002-2023 | 4.79 | No | No | Yes - Off track | Yes | Yes | 2023 |
| Libya | 2002-2023 | 9.57 | No | No | Yes - Off track | Yes | Yes | 2023 |
| Madagascar | 2002-2023 | 5.51 | Yes (2010) | Yes (2017) | Yes - Off track | Yes | No |  |
| Malawi | 2002-2023 | 3.29 | No | No | Yes - Off track | Yes | Yes | 2023 |
| Mali | 2002-2023 | 5.43 | No | No | Yes - Off track | Yes | Yes | 2023 |
| Mauritania | 2002-2023 | 5.94 | No | No | Yes - Off track | Yes | Yes | 2023 |
| Mauritius | 2002-2023 | 8.24 | No | No | Yes - Slow progress | Yes | Yes | 2023 |
| Morocco | 2002-2023 | 10.61 | No | No | Yes - Slow progress | Yes | Yes | 2023 |
| Mozambique | 2002-2023 | 6.76 | No | No | Yes - Off track | Yes | Yes | 2023 |
| Namibia | 2002-2023 | 14.99 | Yes (2002) | Yes (2004) | Yes - Off track | Yes | No |  |
| Niger | 2002-2023 | 6.15 | No | No | Yes - Off track | Yes | Yes | 2023 |
| Nigeria | 2002-2023 | 4.31 | No | No | Yes - Off track | Yes | Yes | 2023 |
| Rwanda | 2002-2023 | 8.92 | No | No | Yes - Off track | Yes | Yes | 2023 |
| Sao Tome and Principe | 2002-2023 | 8.77 | No | No | Yes - Slow progress | Yes | Yes | 2023 |
| Senegal | 2002-2023 | 2.77 | No | No | Yes - Off track | Yes | Yes | 2023 |
| Seychelles | 2002-2023 | 9.58 | No | No | Yes - Off track | Yes | Yes | 2020 |
| Sierra Leone | 2002-2023 | 4.47 | No | No | Yes - Off track | Yes | Yes | 2023 |
| Somalia, Fed. Rep. | 2013-2023 | 7.90 | No | No | No - sensitivity only | Yes | Yes | 2023 |
| South Africa | 2002-2023 | 16.89 | Yes (2015) | Yes (2017) | Yes - Already attained | Yes | No |  |
| South Sudan | 2017-2023 | 2.14 | No | No | No - sensitivity only | No | No |  |
| Sudan | 2002-2023 | 7.15 | No | No | Yes - Off track | Yes | Yes | 2023 |
| Tanzania | 2002-2023 | 5.14 | No | No | Yes - Off track | Yes | Yes | 2023 |
| Togo | 2002-2023 | 2.56 | No | No | Yes - Off track | Yes | Yes | 2023 |
| Tunisia | 2002-2023 | 5.14 | No | No | Yes - Off track | Yes | Yes | 2023 |
| Uganda | 2002-2023 | 4.86 | No | No | Yes - Off track | Yes | Yes | 2023 |
| Zambia | 2002-2023 | 8.89 | No | No | Yes - Off track | Yes | Yes | 2023 |
| Zimbabwe | 2010-2023 | 4.74 | No | No | No - sensitivity only | Yes | Yes | 2023 |

*Notes: The primary trend sample required complete annual outcome observations from 2002 through 2023. Somalia, South Sudan and Zimbabwe are retained in the available data sensitivity analysis. Conditional probabilities are estimated only for countries included in the adjusted Weibull model that had not previously attained.*
