# Supplementary material for: Beyond aspiration: An expenditure-based evaluation of progress towards the Abuja Declaration target and implications for domestic health financing in Africa
Source: PLOS Glob Public Health. 2026 Oct 9;6(10):e0007423. doi: 10.1371/journal.pgph.0007423 (PMC13652233; doi:10.1371/journal.pgph.0007423)
Supplement: Supplementary file 4 [file pgph.0007423.s004.docx]

**S4 Table. Country-specific trend classification after excluding countries with limited outcome variation.**

| **Specification** | **Countries (n)** | **Already attained (n)** | **On track (n)** | **Slow progress (n)** | **Off track (n)** | **Off track (%)** |
| --- | --- | --- | --- | --- | --- | --- |
| Primary complete-history sample | 51 | 1 | 4 | 4 | 42 | 82.4 |
| At least 10 distinct annual values | 50 | 1 | 4 | 4 | 41 | 82.0 |
| At least 15 distinct annual values | 46 | 1 | 4 | 4 | 37 | 80.4 |

**Notes:** The primary specification used 51 countries with complete 2002-2023 outcome histories. The sensitivity specifications retained countries with at least 10 or at least 15 distinct annual outcome values across the 22-year period. These restrictions retained repeated country-years among eligible countries and operated at the country level
