# Supplementary material for: Beyond aspiration: An expenditure-based evaluation of progress towards the Abuja Declaration target and implications for domestic health financing in Africa
Source: PLOS Glob Public Health. 2026 Oct 9;6(10):e0007423. doi: 10.1371/journal.pgph.0007423 (PMC13652233; doi:10.1371/journal.pgph.0007423)
Supplement: Supplementary file 5 [file pgph.0007423.s005.docx]

**S5 Table. Weibull model sample and fit summary.**

| **Model** | **Analytical role** | **Countries (n)** | **Risk intervals (n)** | **First-attainment events (n)** | **Log likelihood** | **Weibull shape parameter** |
| --- | --- | --- | --- | --- | --- | --- |
| Unadjusted Weibull reference | Parsimonious reference | 54 | 1092 | 5 | -22.775 | 0.776 |
| Adjusted Weibull model | Exploratory prediction | 53 | 1064 | 5 | -20.700 | 0.835 |
