# Supplementary material for: Beyond aspiration: An expenditure-based evaluation of progress towards the Abuja Declaration target and implications for domestic health financing in Africa
Source: PLOS Glob Public Health. 2026 Oct 9;6(10):e0007423. doi: 10.1371/journal.pgph.0007423 (PMC13652233; doi:10.1371/journal.pgph.0007423)
Supplement: Supplementary file 6 [file pgph.0007423.s006.docx]

**S6 Table. Adjusted Weibull proportional-hazards estimates.**

| **Model** | **Parameter** | **Coefficient** | **Cluster-robust SE** | **P value** | **Hazard ratio** | **95% CI, lower** | **95% CI, upper** |
| --- | --- | --- | --- | --- | --- | --- | --- |
| Unadjusted Weibull reference | Constant | -4.697 | 1.018 | <0.001 | NA | NA | NA |
| Unadjusted Weibull reference | Log shape parameter | -0.253 | 0.380 | 0.506 | NA | NA | NA |
| Adjusted Weibull model | Log GDP per capita | -0.299 | 0.440 | 0.497 | 0.742 | 0.313 | 1.757 |
| Adjusted Weibull model | Log external health expenditure per capita | 0.111 | 0.289 | 0.701 | 1.117 | 0.634 | 1.969 |
| Adjusted Weibull model | Under-five mortality rate | 0.008 | 0.008 | 0.339 | 1.008 | 0.992 | 1.024 |
| Adjusted Weibull model | Government effectiveness | 1.859 | 0.537 | <0.001 | 6.414 | 2.237 | 18.391 |
| Adjusted Weibull model | Constant | -2.605 | 3.615 | 0.471 | NA | NA | NA |
| Adjusted Weibull model | Log shape parameter | -0.181 | 0.388 | 0.641 | NA | NA | NA |

*Notes: The adjusted model includes log GDP per capita, log external health expenditure per capita, under-five mortality and government effectiveness. Robust standard errors are clustered by country. Only five first-attainment events were observed. The adjusted model and country predictions are exploratory.*
