# Supplementary material for: Beyond aspiration: An expenditure-based evaluation of progress towards the Abuja Declaration target and implications for domestic health financing in Africa
Source: PLOS Glob Public Health. 2026 Oct 9;6(10):e0007423. doi: 10.1371/journal.pgph.0007423 (PMC13652233; doi:10.1371/journal.pgph.0007423)
Supplement: Supplementary file 7 [file pgph.0007423.s007.docx]

**S7 Table. Countries projected to cross the benchmark by 2063 in the primary specification.**

| **Country** | **Government health expenditure, 2023 (% of GGE)** | **Annual change (percentage points)** | **Point-estimate crossing year** | **Probability of crossing by 2063 (%)** | **Conditional 95% simulation interval, lower year** | **Conditional 95% simulation interval, upper year** | **Status** |
| --- | --- | --- | --- | --- | --- | --- | --- |
| South Africa | 16.89 | 0.273 | 2015 | 100.0 | 2015.0 | 2015.0 | Already attained |
| Botswana | 14.61 | 0.349 | 2026 | 100.0 | 2023.0 | 2030.0 | On track |
| Cabo Verde | 13.79 | 0.243 | 2032 | 99.7 | 2024.6 | 2048.4 | On track |
| Lesotho | 11.83 | 0.222 | 2038 | 98.8 | 2029.9 | 2053.5 | On track |
| Gabon | 9.59 | 0.233 | 2046 | 99.6 | 2038.1 | 2056.5 | On track |
| Burkina Faso | 8.78 | 0.204 | 2049 | 89.6 | 2037.1 | 2061.2 | Slow progress |
| Morocco | 10.61 | 0.228 | 2051 | 99.5 | 2045.2 | 2058.7 | Slow progress |
| Sao Tome and Principe | 8.77 | 0.176 | 2055 | 60.7 | 2029.3 | 2061.7 | Slow progress |
| Mauritius | 8.24 | 0.148 | 2058 | 66.4 | 2041.6 | 2062.5 | Slow progress |

*Notes: Crossing-year intervals are conditional on simulated crossing by 2063 and capture coefficient uncertainty under each country-specific linear model. They do not incorporate future policy or structural change.*
