# Supplementary material for: Beyond aspiration: An expenditure-based evaluation of progress towards the Abuja Declaration target and implications for domestic health financing in Africa
Source: PLOS Glob Public Health. 2026 Oct 9;6(10):e0007423. doi: 10.1371/journal.pgph.0007423 (PMC13652233; doi:10.1371/journal.pgph.0007423)
Supplement: Supplementary file 8 [file pgph.0007423.s008.docx]

**S8 Table. Sensitivity of trend classifications to outcome coverage and estimation period.**

| **Specification** | **Countries (n)** | **Already attained (n)** | **On track (n)** | **Slow progress (n)** | **Off track (n)** | **Off track (%)** |
| --- | --- | --- | --- | --- | --- | --- |
| Primary common period, 2002 to 2023 | 51 | 1 | 4 | 4 | 42 | 82.4 |
| Available data, 2002 to 2023 | 54 | 1 | 4 | 4 | 45 | 83.3 |
| Pre-COVID-19 common period, 2002 to 2019 | 51 | 0 | 8 | 3 | 40 | 78.4 |
| Recent period, 2010 to 2023 | 54 | 1 | 9 | 7 | 37 | 68.5 |
