# Supplementary material for: Beyond aspiration: An expenditure-based evaluation of progress towards the Abuja Declaration target and implications for domestic health financing in Africa
Source: PLOS Glob Public Health. 2026 Oct 9;6(10):e0007423. doi: 10.1371/journal.pgph.0007423 (PMC13652233; doi:10.1371/journal.pgph.0007423)
Supplement: Supplementary file 9 [file pgph.0007423.s009.docx]

**S9 Table. System-GMM persistence estimates after restricting outcome variation and excluding exact consecutive-year repeats.**

| **Specification** | **Exact-repeat country-years omitted** | **Lag coefficient** | **Cluster-robust SE** | **95% CI, lower** | **95% CI, upper** | **P value (lag coefficient = 1)** | **Countries (n)** | **Observations (n)** | **AR(2) P value** | **Hansen P value** | **Instruments (n)** |
| --- | --- | --- | --- | --- | --- | --- | --- | --- | --- | --- | --- |
| Primary complete-history sample | 0 | 0.821 | 0.092 | 0.635 | 1.006 | 0.0580 | 51 | 1052 | 0.1741 | 0.9826 | 28 |
| At least 10 distinct annual values | 0 | 0.812 | 0.095 | 0.622 | 1.002 | 0.0521 | 50 | 1043 | 0.1764 | 0.9843 | 28 |
| At least 15 distinct annual values | 0 | 0.813 | 0.094 | 0.623 | 1.003 | 0.0533 | 46 | 959 | 0.2151 | 0.9955 | 28 |
| Exploratory: exact repeated rows excluded | 122 | 0.823 | 0.124 | 0.574 | 1.073 | 0.1609 | 51 | 926 | 0.1300 | 0.9574 | 28 |

**Notes:** The minimum-distinct-value specifications excluded entire countries that did not meet the stated threshold but retained repeated country-years among included countries. The exact-repeat specification returned to the primary 51-country sample and removed individual country-years whose outcome exactly equalled the immediately preceding year's value. Resulting calendar-year gaps remained explicit, so non-adjacent observations were not treated as one-year lags. The reported equality-to-one p-value tests whether the lag coefficient equals one; all four coefficients differed from zero at p<0.001. All specifications used the primary collapsed lag-2-to-3 instrument structure and the same covariates and year effects. AR denotes Arellano-Bond test; CI, confidence interval; GMM, generalised method of moments; and SE, standard error.
