# Supplementary material for: Beyond aspiration: An expenditure-based evaluation of progress towards the Abuja Declaration target and implications for domestic health financing in Africa
Source: PLOS Glob Public Health. 2026 Oct 9;6(10):e0007423. doi: 10.1371/journal.pgph.0007423 (PMC13652233; doi:10.1371/journal.pgph.0007423)
Supplement: Supplementary file 10 [file pgph.0007423.s010.docx]

**S10 Table. Full substantive coefficients from the GMM specifications.**

| **Estimator** | **Parameter** | **Coefficient** | **Cluster-robust SE** | **95% CI, lower** | **95% CI, upper** | **P value** |
| --- | --- | --- | --- | --- | --- | --- |
| One-step difference GMM | Lagged expenditure share | 0.877 | 0.114 | 0.649 | 1.105 | <0.001 |
| One-step difference GMM | Log GDP per capita | -0.835 | 0.825 | -2.491 | 0.821 | 0.3161 |
| One-step difference GMM | Log external health expenditure per capita | -0.306 | 0.110 | -0.526 | -0.086 | 0.0074 |
| One-step difference GMM | Under-five mortality | 0.006 | 0.001 | 0.004 | 0.008 | <0.001 |
| One-step difference GMM | Government effectiveness | 0.221 | 0.851 | -1.487 | 1.928 | 0.7962 |
| Primary two-step system GMM | Lagged expenditure share | 0.821 | 0.092 | 0.635 | 1.006 | <0.001 |
| Primary two-step system GMM | Log GDP per capita | 0.085 | 0.080 | -0.076 | 0.246 | 0.2935 |
| Primary two-step system GMM | Log external health expenditure per capita | -0.025 | 0.050 | -0.125 | 0.074 | 0.6142 |
| Primary two-step system GMM | Under-five mortality | 0.002 | 0.001 | -0.001 | 0.005 | 0.1557 |
| Primary two-step system GMM | Government effectiveness | 0.545 | 0.258 | 0.028 | 1.062 | 0.0393 |
| Restricted-instrument system GMM | Lagged expenditure share | 0.837 | 0.152 | 0.532 | 1.142 | <0.001 |
| Restricted-instrument system GMM | Log GDP per capita | 0.080 | 0.085 | -0.091 | 0.252 | 0.3513 |
| Restricted-instrument system GMM | Log external health expenditure per capita | -0.029 | 0.057 | -0.144 | 0.085 | 0.6101 |
| Restricted-instrument system GMM | Under-five mortality | 0.002 | 0.001 | -0.001 | 0.005 | 0.1638 |
| Restricted-instrument system GMM | Government effectiveness | 0.505 | 0.394 | -0.286 | 1.296 | 0.2060 |

*Notes: All GMM models adjust for log GDP per capita, log external health expenditure per capita, under-five mortality, government effectiveness and year effects. The primary two-step system-GMM model uses collapsed lags 2-3 of the lagged expenditure share as GMM-style instruments and finite-sample-corrected robust standard errors. The restricted model uses collapsed lag 2 only. P-values for coefficients test equality to zero; a confidence interval crossing one for the lag coefficient means complete persistence cannot be rejected*
