# Supplementary material for: Beyond aspiration: An expenditure-based evaluation of progress towards the Abuja Declaration target and implications for domestic health financing in Africa
Source: PLOS Glob Public Health. 2026 Oct 9;6(10):e0007423. doi: 10.1371/journal.pgph.0007423 (PMC13652233; doi:10.1371/journal.pgph.0007423)
Supplement: Supplementary file 11 [file pgph.0007423.s011.docx]

**S11 Table. Lagged expenditure-share estimates and dynamic-panel diagnostics.**

| **Estimator** | **Lag coefficient** | **Cluster-robust SE** | **95% CI, lower** | **95% CI, upper** | **P value (coefficient = 0)** | **Countries (n)** | **Observations (n)** | **AR(1) P value** | **AR(2) P value** | **Hansen P value** | **Instruments (n)** |
| --- | --- | --- | --- | --- | --- | --- | --- | --- | --- | --- | --- |
| Pooled dynamic model + year effects | 0.902 | 0.023 | 0.856 | 0.948 | <0.001 | 51 | 1071 | NA | NA | NA | NA |
| Country and year fixed effects | 0.660 | 0.035 | 0.591 | 0.730 | <0.001 | 51 | 1071 | NA | NA | NA | NA |
| One-step difference GMM | 0.877 | 0.114 | 0.649 | 1.105 | <0.001 | 51 | 1000 | 0.0001 | 0.1703 | 0.8931 | 26 |
| Primary two-step system GMM | 0.821 | 0.092 | 0.635 | 1.006 | <0.001 | 51 | 1052 | 0.0001 | 0.1741 | 0.9826 | 28 |
| Restricted-instrument system GMM | 0.837 | 0.152 | 0.532 | 1.142 | <0.001 | 51 | 1052 | 0.0008 | 0.2316 | 0.8982 | 27 |

**Notes:** AR denotes Arellano-Bond test; CI, confidence interval; GMM, generalised method of moments; NA, not applicable; and SE, standard error. AR and Hansen diagnostics are not applicable to the pooled and fixed-effects estimators
