# Supplementary material for: Beyond aspiration: An expenditure-based evaluation of progress towards the Abuja Declaration target and implications for domestic health financing in Africa
Source: PLOS Glob Public Health. 2026 Oct 9;6(10):e0007423. doi: 10.1371/journal.pgph.0007423 (PMC13652233; doi:10.1371/journal.pgph.0007423)
Supplement: Supplementary file 12 [file pgph.0007423.s012.docx]

**S12 Table. Country- and year-fixed-effects estimates for per-capita PHC and immunisation expenditure.**

| **Outcome** | **Coefficient** | **Cluster-robust SE** | **Coefficient 95% CI, lower** | **Coefficient 95% CI, upper** | **P value** | **Percentage change (%)** | **95% CI for percentage change, lower** | **95% CI for percentage change, upper** | **Countries (n)** | **Observations (n)** |
| --- | --- | --- | --- | --- | --- | --- | --- | --- | --- | --- |
| Government PHC expenditure per capita | 0.142 | 0.022 | 0.098 | 0.186 | <0.001 | 15.3 | 10.3 | 20.4 | 45 | 211 |
| Total immunisation expenditure per capita | 0.051 | 0.066 | -0.083 | 0.185 | 0.4460 | 5.2 | -7.9 | 20.3 | 46 | 199 |
