# Supplementary material for: Beyond aspiration: An expenditure-based evaluation of progress towards the Abuja Declaration target and implications for domestic health financing in Africa
Source: PLOS Glob Public Health. 2026 Oct 9;6(10):e0007423. doi: 10.1371/journal.pgph.0007423 (PMC13652233; doi:10.1371/journal.pgph.0007423)
Supplement: Supplementary file 13 [file pgph.0007423.s013.docx]

**S13 Table. Sensitivity analysis using immunisation expenditure as a percentage of CHE.**

| **Outcome** | **Coefficient** | **Cluster-robust SE** | **Coefficient 95% CI, lower** | **Coefficient 95% CI, upper** | **P value** | **Percentage change (%)** | **95% CI for percentage change, lower** | **95% CI for percentage change, upper** | **Countries (n)** | **Observations (n)** |
| --- | --- | --- | --- | --- | --- | --- | --- | --- | --- | --- |
| Immunisation expenditure as a share of CHE | 0.003 | 0.068 | -0.134 | 0.139 | 0.9694 | 0.3 | -12.5 | 14.9 | 46 | 199 |

Notes: Outcomes are modelled in logarithms. Percentage changes are calculated as 100[exp(beta)-1] for a one-percentage-point increase in government health expenditure as a percentage of GGE. Models include country and year fixed effects with country-clustered standard errors. PHC is domestic government PHC expenditure per capita. Total immunisation expenditure per capita includes all financing sources represented in CHE.
